# Shape Transformation of Polymer Vesicles

**DOI:** 10.1021/accountsmr.3c00253

**Published:** 2024-03-18

**Authors:** Wei Li, Shaohua Zhang, Mingchen Sun, Sandra Kleuskens, Daniela A. Wilson

**Affiliations:** Institute for Molecules and Materials, Radboud University, Heyendaalseweg 135, 6525 AJ Nijmegen, The Netherlands

## Abstract

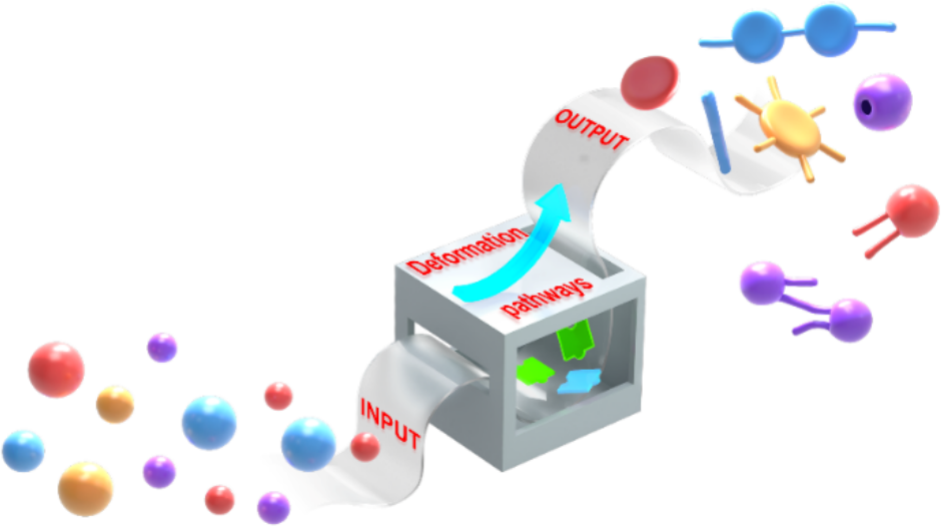

Life activities, such as respiration, are accomplished through the continuous shape modulation of cells, tissues, and organs. Developing smart materials with shape-morphing capability is a pivotal step toward life-like systems and emerging technologies of wearable electronics, soft robotics, and biomimetic actuators. Drawing inspiration from cells, smart vesicular systems have been assembled to mimic the biological shape modulation. This would enable the understanding of cellular shape adaptation and guide the design of smart materials with shape-morphing capability. Polymer vesicles assembled by amphiphilic molecules are an example of remarkable vesicular systems. The chemical versatility, physical stability, and surface functionality promise their application in nanomedicine, nanoreactor, and biomimetic systems. However, it is difficult to drive polymer vesicles away from equilibrium to induce shape transformation due to the unfavorable energy landscapes caused by the low mobility of polymer chains and low permeability of the vesicular membrane. Extensive studies in the past decades have developed various methods including dialysis, chemical addition, temperature variation, polymerization, gas exchange, etc., to drive shape transformation. Polymer vesicles can now be engineered into a variety of nonspherical shapes. Despite the brilliant progress, most of the current studies regarding the shape transformation of polymer vesicles still lie in the trial-and-error stage. It is a grand challenge to predict and program the shape transformations of polymer vesicles. An in-depth understanding of the deformation pathway of polymer vesicles would facilitate the transition from the trial-and-error stage to the computing stage. In this Account, we introduce recent progress in the shape transformation of polymer vesicles. To provide an insightful analysis, the shape transformation of polymer vesicles is divided into basic and coupled deformation. First, we discuss the basic deformation of polymer vesicles with a focus on two deformation pathways: the oblate pathway and the prolate pathway. Strategies used to trigger different deformation pathways are introduced. Second, we discuss the origin of the selectivity of two deformation pathways and the strategies used to control the selectivity. Third, we discuss the coupled deformation of polymer vesicles with a focus on the switch and coupling of two basic deformation pathways. Last, we analyze the challenges and opportunities in the shape transformation of polymer vesicles. We envision that a systematic understanding of the deformation pathway would push the shape transformation of polymer vesicles from the trial-and-error stage to the computing stage. This would enable the prediction of deformation behaviors of nanoparticles in complex environments, like blood and interstitial tissue, and access to advanced architecture desirable for man-made applications.

## 1 Introduction

Smart materials with shape-morphing capability are ubiquitous in living organisms and have been the cornerstone in emerging technologies of soft robotics, flexible electronics, and biomimetic actuators.^1,2^ The biological membrane is a remarkable example, which renders cells the unique capability to adapt their shapes for membrane trafficking, cellular migration, and cellular division.^3^ These fantastic functions stimulate the development of smart vesicular systems to mimic cellular shape adaptation. For cells, shape transformation is accomplished through the dynamic reconfiguration of lipid molecules within biological membranes, driven by a multitude of shape-modulating factors, including the cytoskeleton, membrane-bending proteins, coat proteins, etc.^4^ For smart vesicular systems, significant progress has been witnessed by encapsulating biological shape-modulating factors into artificial vesicles to induce shape transformation.^5^ In the last two decades, there has been a notable proliferation of artificial vesicles, from lipid to synthetic amphiphiles, such as dendrimers and polymer vesicles, aiming to extend their chemical and physical limits.^6−8^ The high energy cost in reconfiguring synthetic amphiphiles within vesicular membranes compromised the efficacy of biological shape-modulating factors and thus necessitated the development of new strategies for shape transformation.^9^

**Figure 1:**
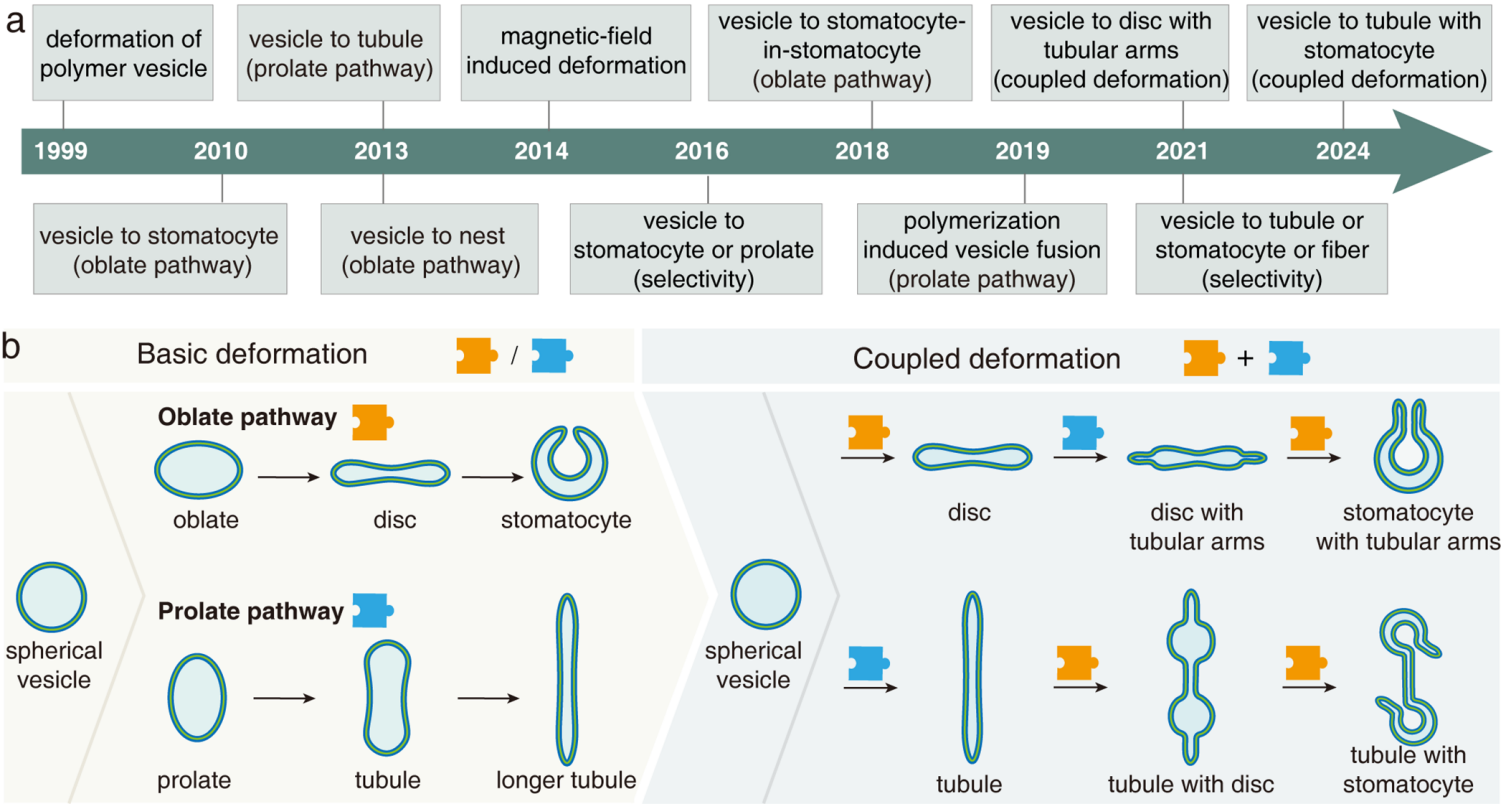
(a) Timeline of the shape transformation of polymer vesicles. (b) Basic deformation and coupled deformation of polymer vesicles. In basic deformation, the vesicle is transformed into the stomatocyte through the oblate pathway or a tubule through the prolate pathway. In coupled deformation, the vesicle is transformed into the disc/stomatocyte with tubular arms or a tubule with disc/stomatocyte through the coupling of oblate and prolate pathways.

Polymer vesicles represent a noteworthy category of artificial vesicles, holding great promise for nanomedicine, nanoreactors, and biomimetic systems due to their chemical versatility and physical stability.^10^ The shape of polymer vesicles is recognized as a crucial parameter in dictating their circulation time, cellular uptake efficiency, and targeting capability when employed as drug carriers.^11−13^ The engineering of polymer vesicles to exhibit adaptive shapes can facilitate the development of smart drug delivery systems. Research on the shape transformation of polymer vesicles can be dated back to 1999 and broadly divided into two developmental phases (Figure 1a). In the initial phase spanning from 1999 to 2009, shape transformation of polymer vesicles was successfully induced without the use of biological shape-modulating factors, for instance, transformation from a tubule to a vesicle by osmotic pressure, tubule generation by optical tweezers, and destruction of a vesicle by light irradiation (Table 1).^7,14−16^ The subsequent phase starting in 2010 witnessed the emergence of controlled shape transformation and the identification of different deformation pathways (Figure 1b).^17−37^ Spherical vesicles have been transformed into a variety of nonspherical shapes including a disc, stomatocyte (sto), nest, sto-in-sto, tubule, disc with tubular arms, tubule with sto, etc. Although more and more shapes have been accessed, shape transformation of polymer vesicles still lies in the trial-and-error stage. It is a grand challenge to compute the output shapes according to the input parameters. We envision that a systematic understanding of the deformation pathway of polymer vesicles would facilitate the transition from the trial-and-error to the computing stage.

**Table 1:**
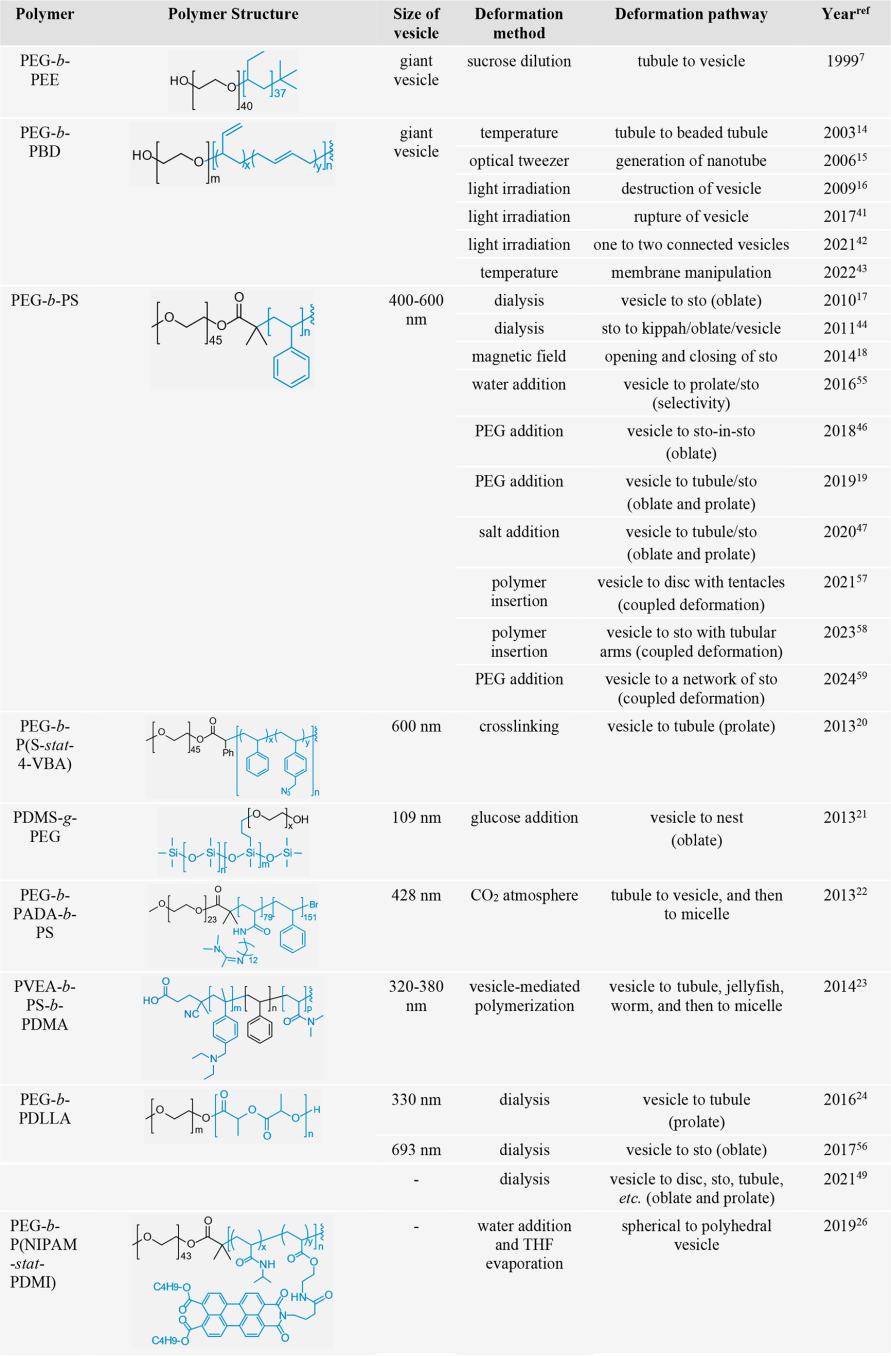

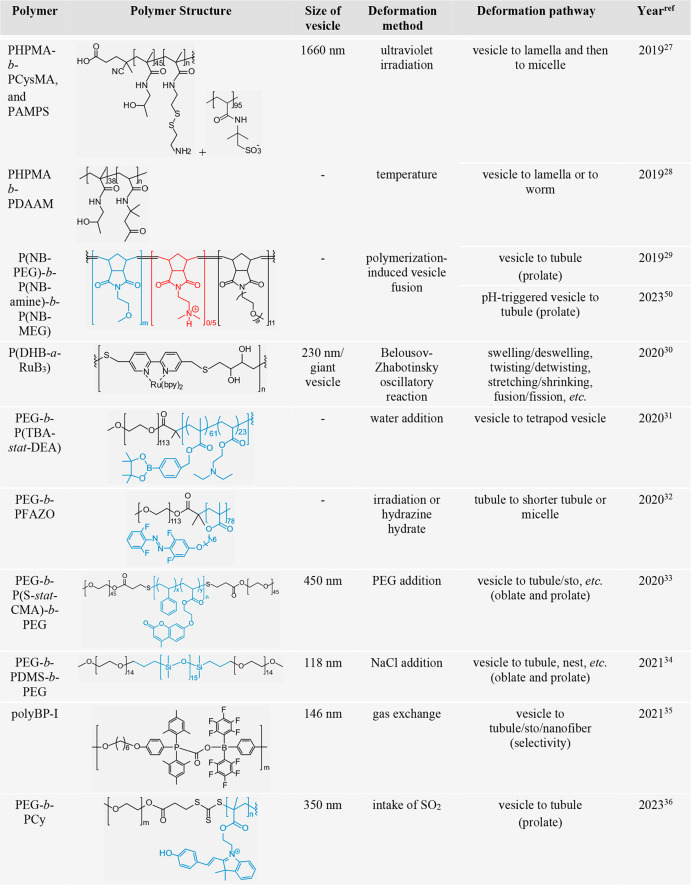
Examples of the Shape Transformation of Polymer Vesicles^a^

a PEG, poly(ethylene glycol); PEE, poly(ethyl ethylene); PBD, poly(butadiene); PS, polystyrene; P(4-VBA), poly(4-vinylbenzyl azide); PDMS, polydimethylsiloxane; PADA, poly((*N*-amidine)dodecylacrylamide); PVEA, poly(*N*-(4-vinylbenzyl)-*N*,*N*-diethylamine); PDMA, poly(*N*,*N*-dimethylacrylamide); PDLLA, poly(d,l-lactide); P(NIPAM), poly(*N*-isopropylacrylamide); P(PDMI), poly(perylene diester monoimide); PHPMA, poly(2-hydroxypropyl methacrylamide); PCysMA, poly(cystamine methacrylamide hydrochloride); PAMPS, poly(2-acrylamido-2-methylpropanesulfonic acid); PDAAM, poly(diacetone acrylamide); P(NB-PEG), poly(*exo*-norbornene imide poly(ethylene glycol) methyl ether); P(NB-amine), poly(*exo*-norbornene imide tertiary amine); P(NB-MEG), poly(*exo*-norbornene imide ethylene glycol monomethyl ether); P(DHB), poly(2,3-dihydroxybutylene); P(RuB_3_), poly(bipyridyl-dithioether-dichloro-bis(bipyridine)ruthenium); P(TBA), poly(4-(4,4,5,5-tetramethyl-1,3,2-dioxaborolan-2-yl)benzyl methacrylate); P(DEA), poly(2-(diethylamino)ethyl methacrylate); PFAZO, poly(6-(4-((2,6-difluorophenyl) diazenyl)-3,5-difluorophenoxy) hexyl methacrylate); P(CMA), poly(coumarin methacrylate); PCy, poly(2-hydroxyphenyl cyanine); *b*, block; *g*, graft; *a*, alternating; *stat*, statistical; sto, stomatocyte. Vesicle indicates a spherical architecture without special notification.

In this Account, we provide our insights into the shape transformation of polymer vesicles. To deepen the understanding of the deformation pathway, the shape transformation of polymer vesicles is divided into basic deformation and coupled deformation (Figure 1b). The basic deformation of polymer vesicles, selectivity of the deformation pathway, and coupled deformation of polymer vesicles are discussed in sequence by referring to the excellent results of the literature and the work of our group. Finally, we analyzed the challenges and opportunities in the shape transformation of polymer vesicles, trying to identify the future direction. The preparation of nanoparticles with various morphologies through macromolecular self-assembly will not be discussed in this Account. We refer the readers to recent reviews on this subject for more information.^38−40^

## 2 Basic Deformation of Polymer Vesicles

### 2.1 Oblate Pathway

According to the seminal works in the past decades, shape transformation of polymer vesicles can be briefly divided into basic deformation and coupled deformation (Figure 1b). Basic deformation mainly includes two deformation pathways: (I) oblate pathway, e.g., vesicle to oblate, disc, stomatocyte; (II) prolate pathway, e.g., vesicle to prolate, tubule. Coupled deformation is enabled through the sequential coupling of two basic deformation pathways. Besides the above-mentioned pathway, there also exist other deformation pathways, such as vesicle to micelle, rupture of the vesicle, formation of the polyhedral vesicle, etc.^25,41−43^ These deformation pathways will not be discussed in this Account.

**Figure 2:**
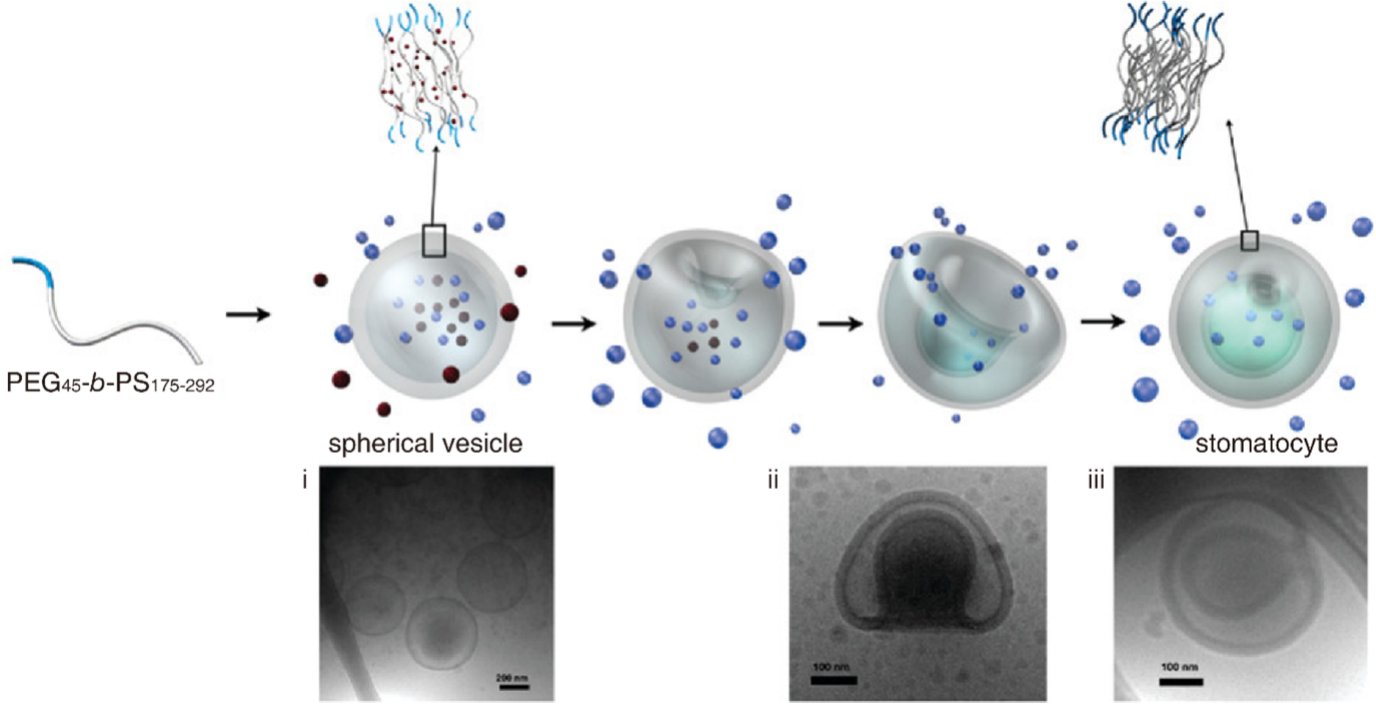
Oblate pathway. Shape transformation from vesicle to stomatocyte induced by dialysis against water. Cryo-TEM images of the vesicle (i), stomatocyte with a big opening (ii), and stomatocyte with a small opening (iii). Reproduced with permission from ref (17). Copyright 2010 American Chemical Society.

The oblate pathway was first reported by van Hest and co-workers in 2010 (Figure 2).^17^ The polymer vesicle assembled by poly(ethylene glycol)-*block*-polystyrene (PEG-*b*-PS) is transformed into the stomatocyte through this pathway. Shape transformation is induced by dialyzing the dispersion of polymer vesicles in a mixture of 50 vol % water and 50 vol % organic solvents (THF:dioxane = 1:1 v/v) against pure water. The organic solvent acts as a plasticizer to retain the mobility of PS chains and the permeability of the vesicular membrane. During dialysis, the osmotic gradient generated by the different solvent compositions across the vesicular membrane induces the rapid expulsion of solvent held within the lumen of polymer vesicles through the plasticized vesicular membrane. The diminished volume of the lumen is proposed to drive the transformation from vesicle to stomatocyte through the inward folding of the vesicular membrane. When the organic solvent is gradually introduced back into the system, the stomatocyte can be transformed back to oblate or vesicle by refilling the lumen.^44^ The above research contributes to the first demonstration of the oblate pathway for polymer vesicles.

The benefit of using PEG-*b*-PS vesicles for shape transformation is that their intermediate shapes can be captured by vitrifying PS chains of the vesicular membrane through the addition of an excess amount of water, allowing researchers to investigate the deformation pathway. Nevertheless, the vitrification of PS would lead to diminished chain mobility and membrane permeability when the dispersion of polymer vesicles is dialyzed against water, which would inhibit the further deformation of the stomatocyte, thus leaving a large opening (Figure 2). To retain the flexibility of PS chains for a longer time, the percentage of THF, a better plasticizer of PS than dioxane, in the organic solvent of the dispersion is increased. With the increase of THF percentage, the stomatocyte with a smaller opening is obtained, suggesting the successful triggering of the further shape transformation (Figure 2iii). When the THF percentage reaches 90 vol %, the opening can be completely closed.^45^ While further deformation is possible, the stomatocyte remains the outcome of the oblate pathway triggered by the dialysis method.

**Figure 3:**
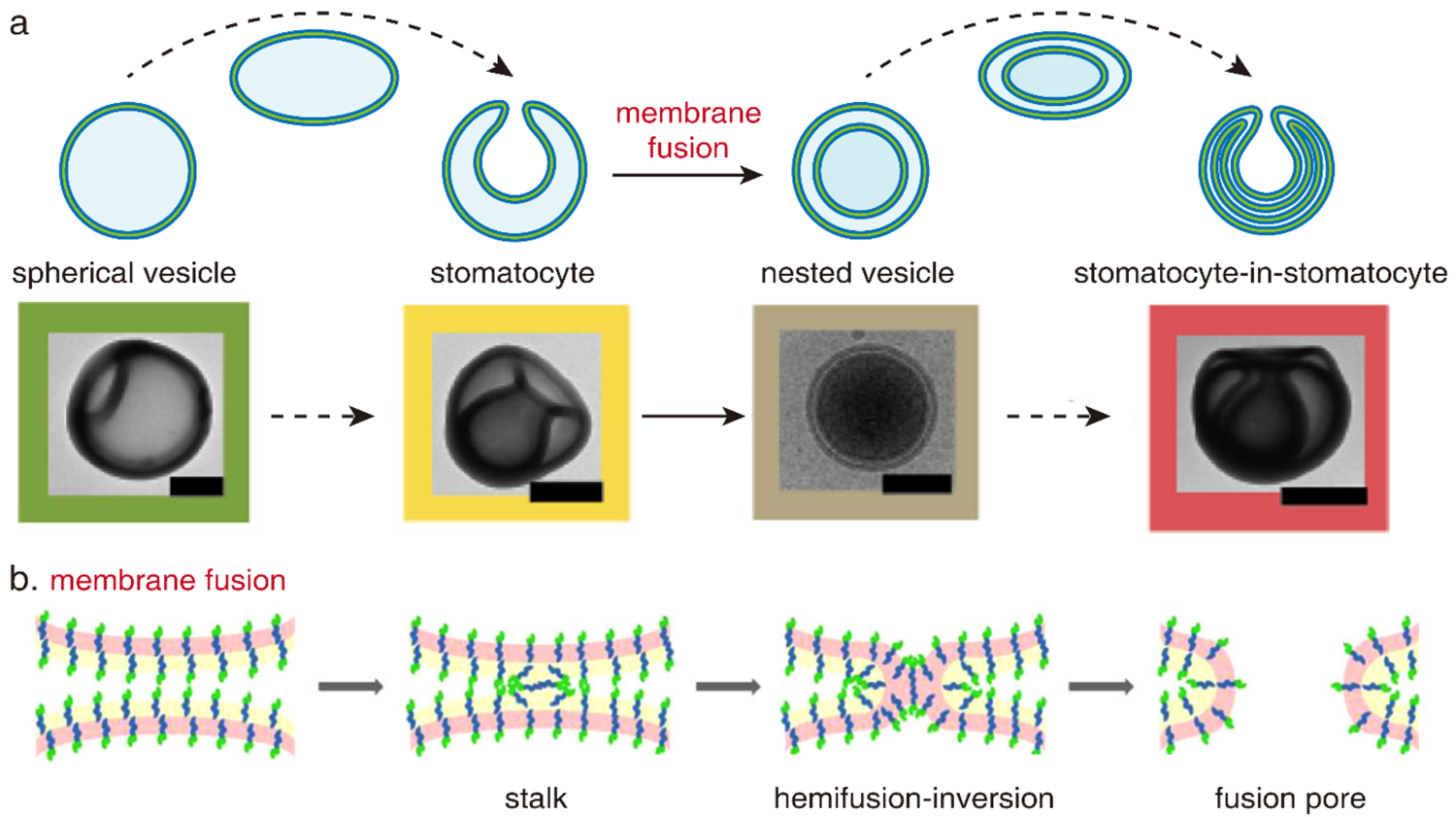
Oblate pathway with membrane fusion. (a) Shape transformation from vesicle to stomatocyte-in-stomatocyte by chemical addition. TEM images of vesicle, stomatocyte, nest, and stomatocyte-in-stomatocyte are shown. Scale bar is 200 nm. Reproduced with permission from ref (46). Copyright 2018 American Chemical Society. (b) Fusion of the vesicular membrane formed by PDMS-*g*-PEG. Reproduced with permission from ref (21). Copyright 2013 American Chemical Society.

To explore the further transformation along the oblate pathway, the chemical addition method was developed. Poly(ethylene glycol) with a molecular weight of 2000 Da (PEG2000) or salt is added into the dispersion of vesicles to induce shape transformation.^19,46−48^ In comparison to dialysis, chemical addition only slightly alters the ratio of organic solvent in the dispersion, thereby preserving the flexibility of PS chains. The added PEG2000 stays in the outside of vesicles and results in an osmotic gradient across the vesicular membrane, which drives the shape transformation of polymer vesicles. Under low PEG2000 concentration, the vesicle is transformed into the stomatocyte through the oblate pathway. With the increase of PEG2000 concentration, the vesicle is transformed to vesicle-in-vesicle (nest) and sto-in-sto (Figure 3a), demonstrating the further transformation along the oblate pathway.^46^ The transformation from stomatocyte to nest is caused by the fusion of the vesicular membrane at the opening of the stomatocyte. The transformation from nest to sto-in-sto is driven by the osmotic gradient. The high PEG2000 concentration is anticipated to contribute to membrane fusion through the depletion effect and water bonding and provide enough driving force for further shape transformation. However, if the concentration of PEG2000 is too high, the large compound vesicle is obtained due to the uncontrolled fusion of the vesicular membrane.^19^ The chemical addition method unravels the further transformation of polymer vesicles along the oblate pathway.

Fusion of the vesicular membrane plays a vital role in the oblate pathway. Lecommandoux and co-workers proposed that the membrane fusion adopts the hemifusion–inversion step by using vesicles assembled by polydimethylsiloxane-*graft*-poly(ethylene glycol) (PDMS-*g*-PEG) as the model (Figure 3b).^21^ During shape transformation, the vesicular membrane at the opening of the stomatocyte would first approach each other (stalk). The polymer chains then rearrange themselves for hemifusion–inversion. Finally, the vesicular membrane separates from each other, forming two distinct vesicles. The bilayer structure of the vesicular membrane allows fusion to occur through subtle adjustments in the conformation of polymer chains. As vesicles are enclosed by a monolayer of triblock copolymer, membrane fusion is impeded due to the unfavorable hairpin conformation formed during hemifusion–inversion. Membrane fusion extends the oblate pathway, enhancing the diversity of shapes achievable by polymer vesicles.

### 2.2 Prolate Pathway

**Figure 4:**
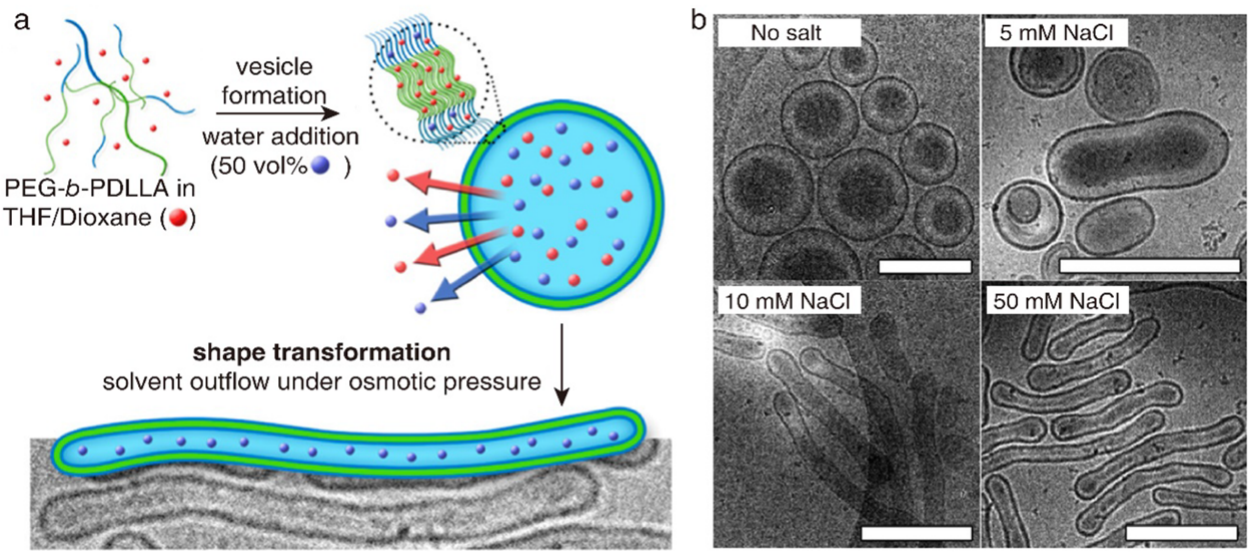
Prolate pathway. (a) Shape transformation from the vesicle to the tubule induced by dialysis against NaCl solution at 4 °C. (b) Cryo-TEM images of polymer assemblies obtained by shape transformation. The vesicle is assembled with PEG_22_-*b*-PDLLA_45_. Reproduced with permission from ref (24). Copyright 2016 American Chemical Society.

Besides the oblate pathway, the polymer vesicle can also be transformed into the tubule through the prolate pathway. The first observation of the prolate pathway in polymer vesicle can be dated back to 1999 when a giant vesicle assembled by poly(ethylene glycol)-*block*-poly(ethyl ethylene) (PEG-*b*-PEE) was transformed to tubule driven by the osmotic gradient of sucrose.^7^ This highlights the capability of the osmotic gradient in driving different deformation pathways. Our group systematically investigated the prolate pathway driven by an osmotic gradient.^24,49^ Vesicles assembled by poly(ethylene glycol)-*b*-poly(d,l-lactide) (PEG_22_-*b*-PDLLA_45_) are dispersed in a mixture of 50 vol % water and 50 vol % organic solvent (THF:dioxane = 4:1 v/v), which is then dialyzed against NaCl solution (Figure 4). In the absence of NaCl, PEG-*b*-PDLLA vesicles maintain the spherical structure throughout the dialysis process. With the increase of NaCl concentration, PEG-*b*-PDLLA tubules are obtained through the prolate pathway. This stands in sharp contrast to the transformation along the oblate pathway observed for PEG-*b*-PS vesicles. Here, NaCl is proposed to contribute not only to the increased osmolarity but also to the asymmetry of the vesicular bilayer membrane (spontaneous curvature), which together triggers the prolate pathway. Further investigation confirmed the impact of spontaneous curvature on the deformation pathway of polymer vesicles by manipulating the structure of PEG-*b*-PDLLA and the composition of the organic solvent.^49^

Besides the osmotic gradient, chemical modification can also trigger the prolate pathway. Van Hest and co-workers reported the shape transformation along the prolate pathway induced by chemical cross-linking.^20^ Specifically, the vesicle assembled by poly(ethylene glycol)-*block*-poly(styrene-*stat*-4-vinylbenzyl azide) (PEG-*b*-P(S-*stat*-4-VBA)) is transformed into the tubule by cross-linking the vesicular membrane with strain-promoted alkyne–azide cycloaddition through the addition of cross-linker. The elongation of vesicles shows dependence on the molar ratio of cross-linker and azide in the vesicular membrane. The vesicle would retain a spherical shape at a molar ratio of 1:1 and begin to elongate when the molar ratio is up to 2:1. Considering the low permeability of the vesicular membrane, the outer layer of the vesicular membrane is easier to cross-link. The varied cross-linking density is expected to result in the asymmetry of the bilayer membrane, which triggers the prolate pathway. More recently, Yan and co-workers reported SO_2_-driven elongation of polymer vesicles.^36^ The cyanine group attached to the hydrophobic segment of amphiphilic block copolymers is converted into 3*H*-indole by reacting with SO_2_. This conversion elevates the hydrophilicity of the hydrophobic segment, alleviating the surface tension of the vesicular membrane. Moreover, π–π interaction between cyanine is replaced by stronger hydrogen-bonding interaction between 3*H*-indole, which diminished the stretching of the hydrophobic segment. The balance between surface tension and stretching degree drives the shape transformation along the prolate pathway.

**Figure 5:**
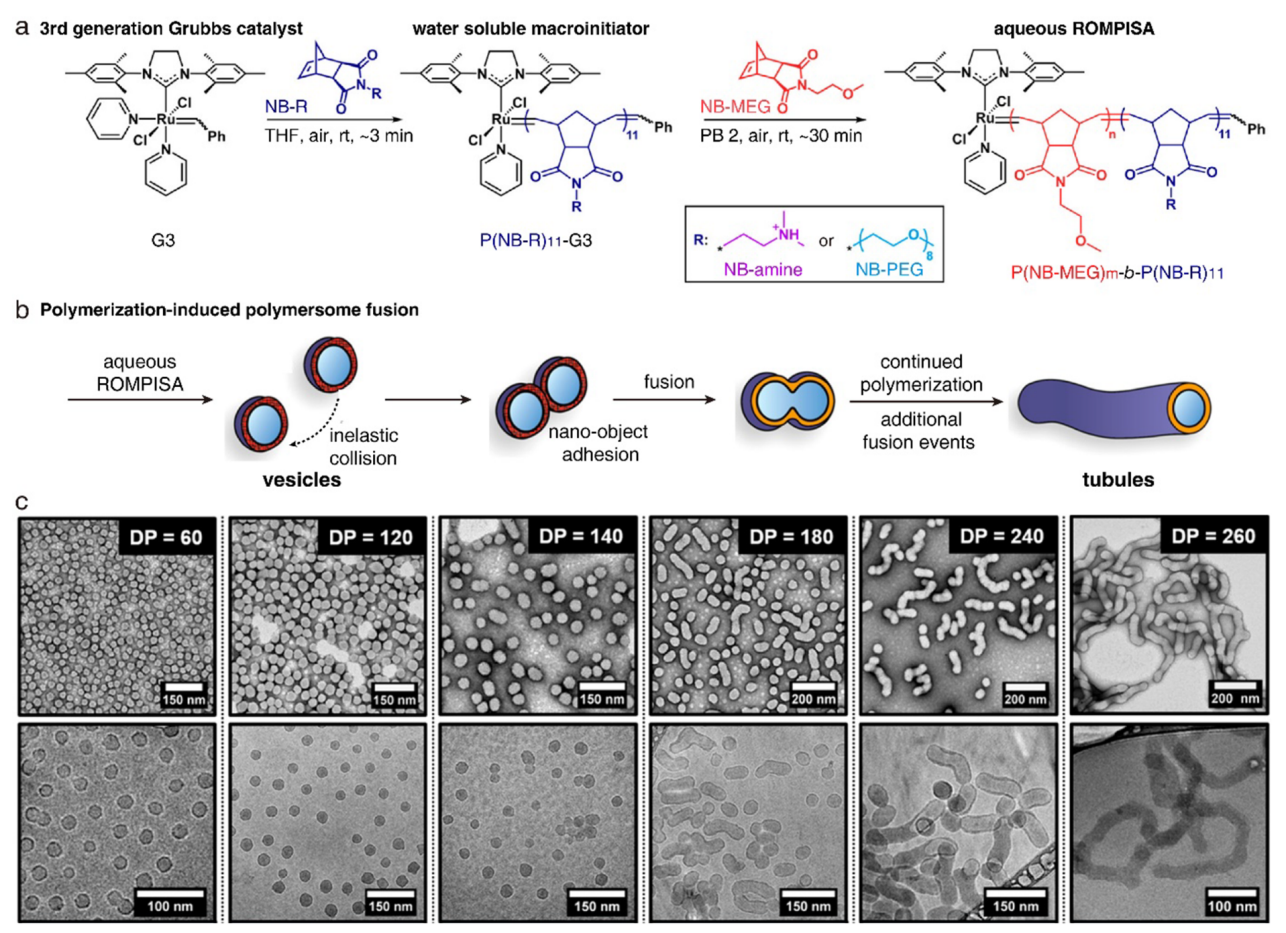
Prolate pathway with membrane fusion. (a) Synthesis of P(NB-R)_11_-*b*-P(NB-MEG)_*m*_ by aqueous ring-opening metathesis polymerization. (b) Shape transformation from the vesicle to the tubule induced by polymerization-induced vesicle fusion. (c) TEM (top row) and cryo-TEM (bottom row) images of polymer assemblies obtained at different degrees of polymerization (DP). Reproduced with permission from ref (29). Copyright 2019 American Chemical Society.

In the traditional prolate pathway, the tubule is formed through the deformation of a single vesicle. Recent research has shown that a tubule can also be obtained through the fusion of multiple vesicles during polymerization-induced self-assembly (Figure 5).^29,50−54^ The fusion of vesicles typically requires meeting the following criteria: (1) the vesicles can adhere to one another, retaining close contact; (2) the membrane tension is sufficient for surpassing the energy barriers associated with fusion; (3) a limited degree of chain mobility of the core-forming block. O’Reilly and co-workers reported that the continuous growth of the hydrophobic core in the vesicular membrane can build up membrane tension, driving the fusion of vesicles.^29^ Specifically, they synthesized a block copolymer consisting of poly(*exo*-norbornene imide poly(ethylene glycol) methyl ether) (P(NB-PEG)) as the hydrophilic segment and poly(*exo*-norbornene imide ethylene glycol monomethyl ether) (P(NB-MEG)) as the hydrophobic segment through aqueous ring-opening metathesis polymerization (Figure 5a). When the polymerization degree of P(NB-MEG) falls within the range of 40–120, the polymers are initially assembled into vesicles. With the increase of polymerization degree, vesicles are fused into tubules (Figure 5c). The growth of P(NB-MEG) is proposed to contribute to the fusion by building membrane tension. In a follow-up work, they reported pH-triggered vesicular fusion by incorporating poly(*exo*-norbornene imide tertiary amine) (P(NB-amine)) between P(NB-PEG) and P(NB-MEG).^50^ The protonation of tertiary amine of P(NB-amine) would render vesicles with a positive charge, which inhibits vesicular fusion by presenting charge repulsion. When tertiary amine is deprotonated by adjusting the pH, vesicles are fused into tubules. The polymerization-induced vesicular fusion opens up new possibilities for shape transformation along the prolate pathway.

The fusion of vesicles results not only in one-dimensional tubules but also in three-dimensional architectures. Du and co-workers reported the formation of tetrapod vesicles induced by water addition.^31^ Vesicles assembled by poly(ethylene glycol)-*block*-poly(4-(4,4,5,5-tetramethyl-1,3,2-dioxaborolan-2-yl)benzyl methacrylate-*stat*-2-(diethylamino)ethyl methacrylate) (PEG-*b*-P(TBA-*stat*-DEA)) are used for the demonstration. During the addition of water, the vesicles are fused into dipod, tripod, and tetrapod vesicles. It is proposed that the relatively rigid TBA acts as the profusion moiety, and the flexible DEA acts as the antifusion moiety of the vesicular membrane. The fusion is induced by the imbalance of the profusion and antifusion force caused by water addition.

## 3 Selectivity of the Deformation Pathway

**Figure 6:**
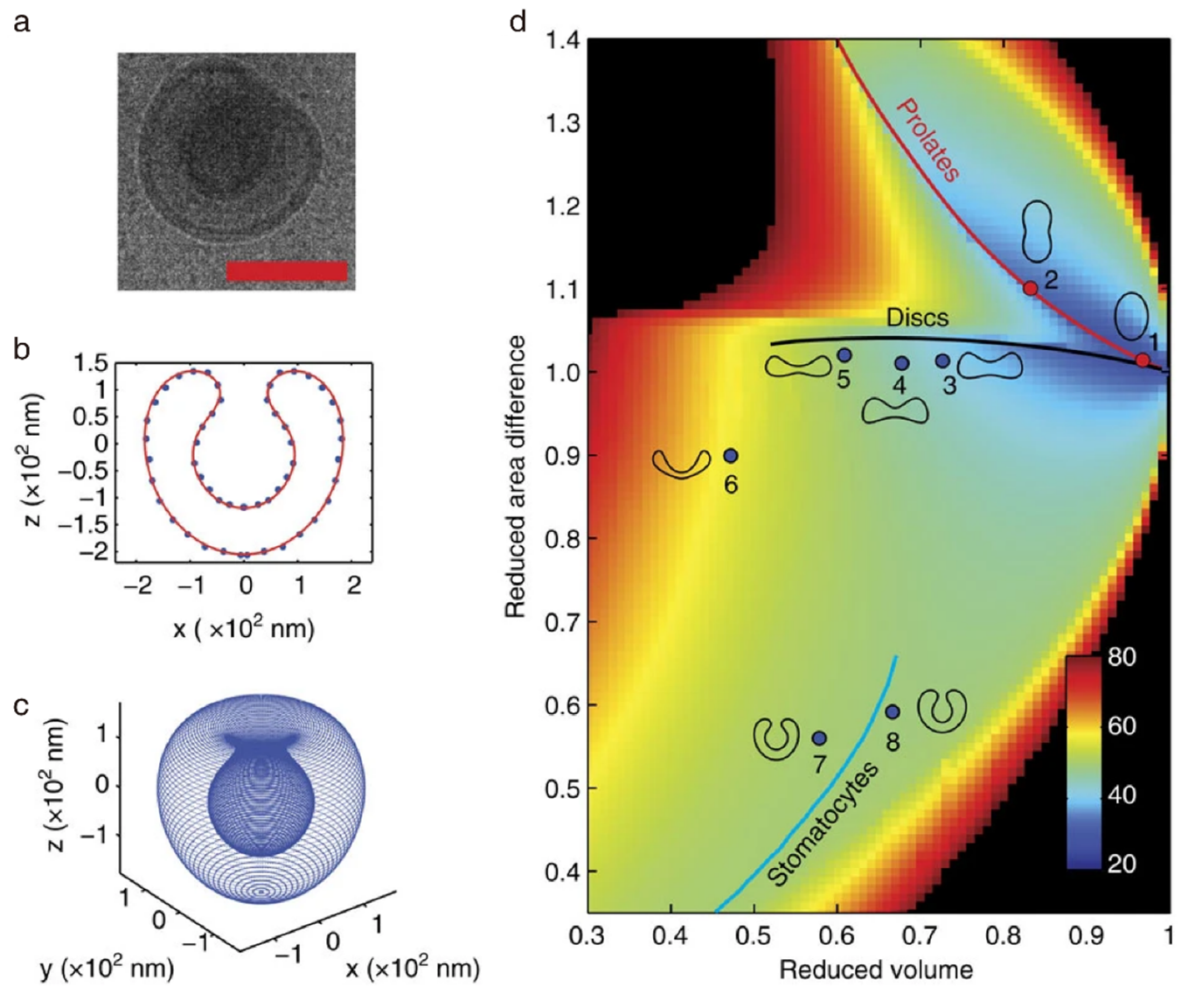
Basic deformation pathways hinted by minimized bending energy. (a–c) Parameterization and reconstruction of stomatocyte based on cryo-TEM images. (d) Phase diagram depicting the position of shapes obtained by shape transformation. The color scale of the background represents the minimized bending energy calculated using the spontaneous curvature model with zero spontaneous curvature. The line indicates local minima for prolate (red line), oblate (black line), and stomatocyte (blue line). Reproduced with permission from ref (55). Copyright 2016 Springer Nature.

After triggering the oblate and prolate pathways, our focus shifted to exploring the selectivity within the deformation pathway.^55^ Water is injected into the dispersion of PEG-*b*-PS vesicles in a mixture of water and organic solvent to create a varied solvent composition across the vesicular membrane. The generated osmotic gradient is used to control the deformation pathway of polymer vesicles. When the water ratio reaches 50 vol %, the prolate pathway is triggered. However, when the water ratio reaches 75 vol %, the oblate pathway is triggered. The obtained shapes are parametrized to elucidate the origin of different deformation pathways, where the reduced volume (*v*) and the reduced area difference (Δ*a*) between the outer and the inner layers of the vesicular membrane are calculated (Figure 6a–c). *v* represents the extent of deflation. Δ*a* serves as a free parameter to distinguish different shapes. These two parameters determine the position of a shape in the phase diagram (Figure 6d). The color scale in the background denotes the minimized bending energy, which is the energy required to deform the membrane, calculated using the spontaneous-curvature model with a zero spontaneous curvature. Trajectories of local minima as a function of *v* are delineated with solid lines. We found that the shape obtained by the oblate and prolate pathways aligns well with the corresponding lines, suggesting that the basic deformation pathways are favored by the minimum bending energy. Besides solvent composition, the type of ions in the dispersion of vesicles is also shown to impact the deformation pathway.^47^ For instance, PEG-*b*-PS vesicles would transform along the prolate pathway in the presence of 0.01 M SCN^–^ or NO_3_^–^ while along the oblate pathway under the same concentration of Cl^–^. The impact of cation species on regulating the deformation pathway is not apparent. Our work highlights that the same polymer vesicles can be adapted to transform along different pathways responding to the external environment.

**Figure 7:**
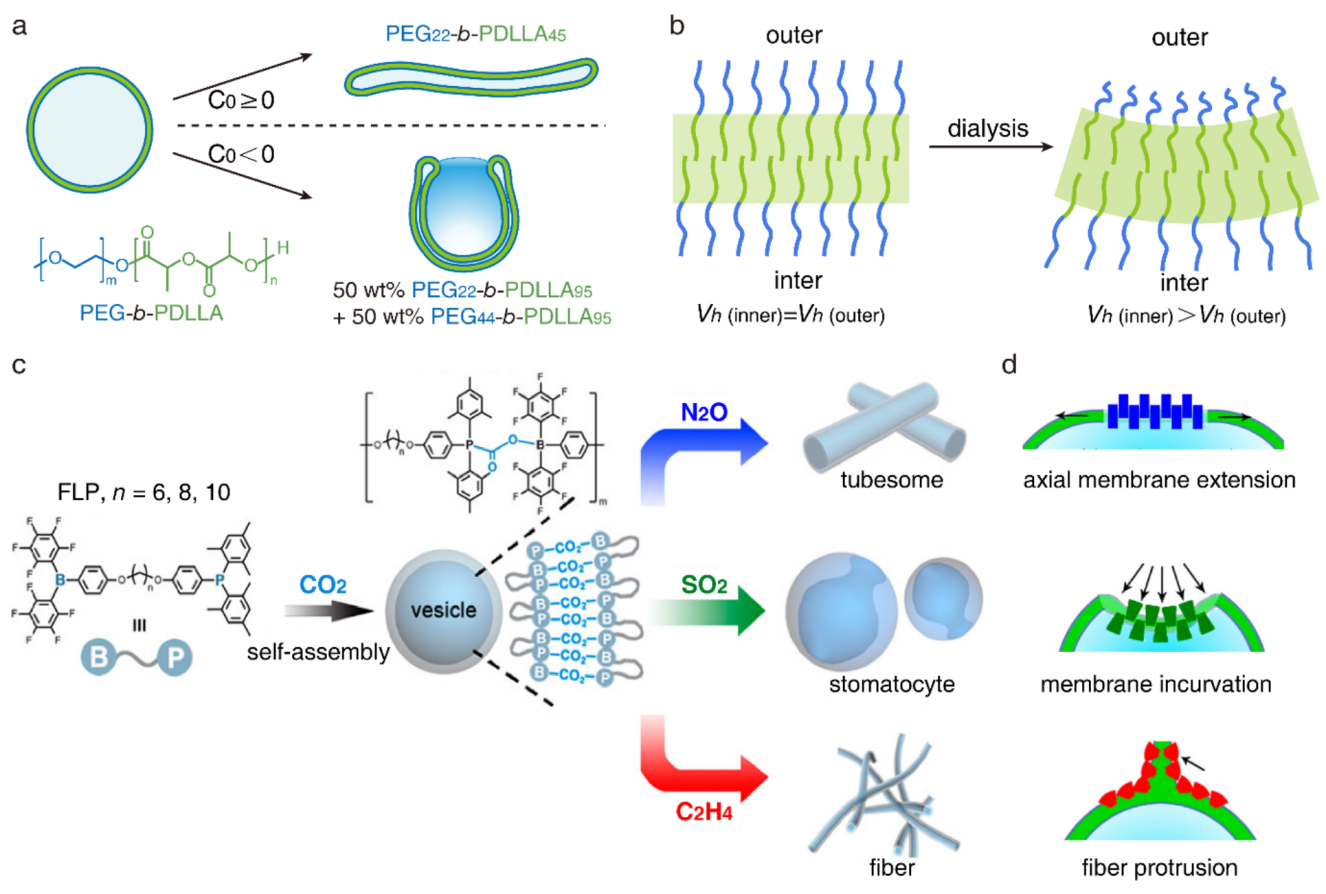
Selectivity of basic deformation pathways. (a) Shape transformation from vesicle to tubule through the prolate pathway or to stomatocyte through the oblate pathway. Selectivity is enabled by manipulating the chemical structure of PEG-*b*-PDLLA. Reproduced with permission from ref (56). Copyright 2017 American Chemical Society. (b) Schematic generation of negative spontaneous curvature by dialysis against NaCl solution. *V*_h_ is the hydrodynamic chain volume. (c) Shape transformation from the vesicle to the tubule, stomatocyte, or fiber through gas exchange. Selectivity is enabled by manipulating the chemical structure of the gas-connection unit. (d) Deformation of the vesicular membrane induced by rearrangement of gas-connection unit. Reproduced with permission from ref (35). Copyright 2021 American Chemical Society.

The selectivity of the deformation pathways is also achieved by manipulating the chemical structure of PEG-*b*-PDLLA vesicles.^56^ When dialyzed against NaCl solution, vesicles assembled by PEG_22_-*b*-PDLLA_45_ would like to transform along the prolate pathway while polymer vesicles assembled by 50 wt % PEG_22_-*b*-PDLLA_95_ and 50 wt % PEG_44_-*b*-PDLLA_95_ would like to transform along the oblate pathway (Figure 7a). In general, a decrease in the dimension of the outer layer of the vesicular membrane will promote a negative curvature, favoring the oblate pathway, while a decrease in the dimension of the inner layer will promote a positive curvature, favoring the prolate pathway.^49^ During dialysis, organic solvent outside of the vesicles is diluted by water, which leads to the collapse of PEG chains and a reduction of the dimension of the outer layer (Figure 7b). Therefore, vesicles assembled by 50 wt % PEG_22_-*b*-PDLLA_95_ and 50 wt % PEG_44_-*b*-PDLLA_95_ transform along the oblate pathway. For vesicles assembled by PEG_22_-*b*-PDLLA_45_, it is proposed that the collapse of short PEG_22_ chains cannot induce a substantial decrease in dimension, thus leading to the prolate pathway. Overall, the deformation pathway is controlled by manipulating the size of PEG chains in the vesicular membrane.

Yan and co-workers showed that the deformation pathway can be controlled by gas exchange.^35^ The heteroditopic frustrated Lewis pair (FLP) monomer is designed by linking triarylborane and triarylphosphine with alkyl chains (Figure 7c). CO_2_ is selected as the gas-connection unit to initiate the assembly of FLP into the vesicles. The replacement of CO_2_ with other gas molecules would induce the shape transformation of polymer vesicles. N_2_O treatment would trigger the prolate pathway, SO_2_ treatment would trigger the oblate pathway, and C_2_H_4_ would induce the transformation of vesicles to fibers. The authors proposed that the geometry of the gas-connection unit controls the deformation pathway (Figure 7d). The N_2_O-connection unit exhibits a column structure, which induces the axial membrane extension and triggers the prolate pathway. C_2_H_4_-connected polymers exhibit a short-wedge structure, which induces fiber protrusion and enables the transformation from vesicle to fiber. The SO_2_-connection unit possesses a similar structure to the CO_2_-connection unit. Nevertheless, the high polarity for the SO_2_-connection unit would reduce the permeability of the vesicular membrane to solvents, thereby increasing the osmotic imbalance across the vesicular membrane, which triggers the oblate pathway. The gas exchange presents a new strategy to control the deformation pathway of polymer vesicles.

## 4 Coupled Deformation of Polymer Vesicles

### 4.1 Sequential Coupling of the Oblate and Prolate Pathways by Polymer Insertion

Guided by the understanding of selectivity, we investigated the coupling of the basic deformation pathways.^57−59^ We believe that the coupling of deformation pathways would facilitate the advent of the computing stage for the shape transformation of polymer vesicles. Coupled shape transformation refers to the simultaneous existence of two basic deformation pathways, which thus requires spatiotemporal control over the deformation process. Cells are remarkable in regulating the deformation of biological membranes by modulating their interactions with biological machinery.^3^ For instance, amphipathic proteins can be inserted into biological membranes to induce the generation of nanoscopic curvature at a specific location. Nevertheless, it is difficult to induce local deformation of polymer vesicles with synthetic machinery due to the poor control over its interaction with the vesicular membrane.

**Figure 8:**
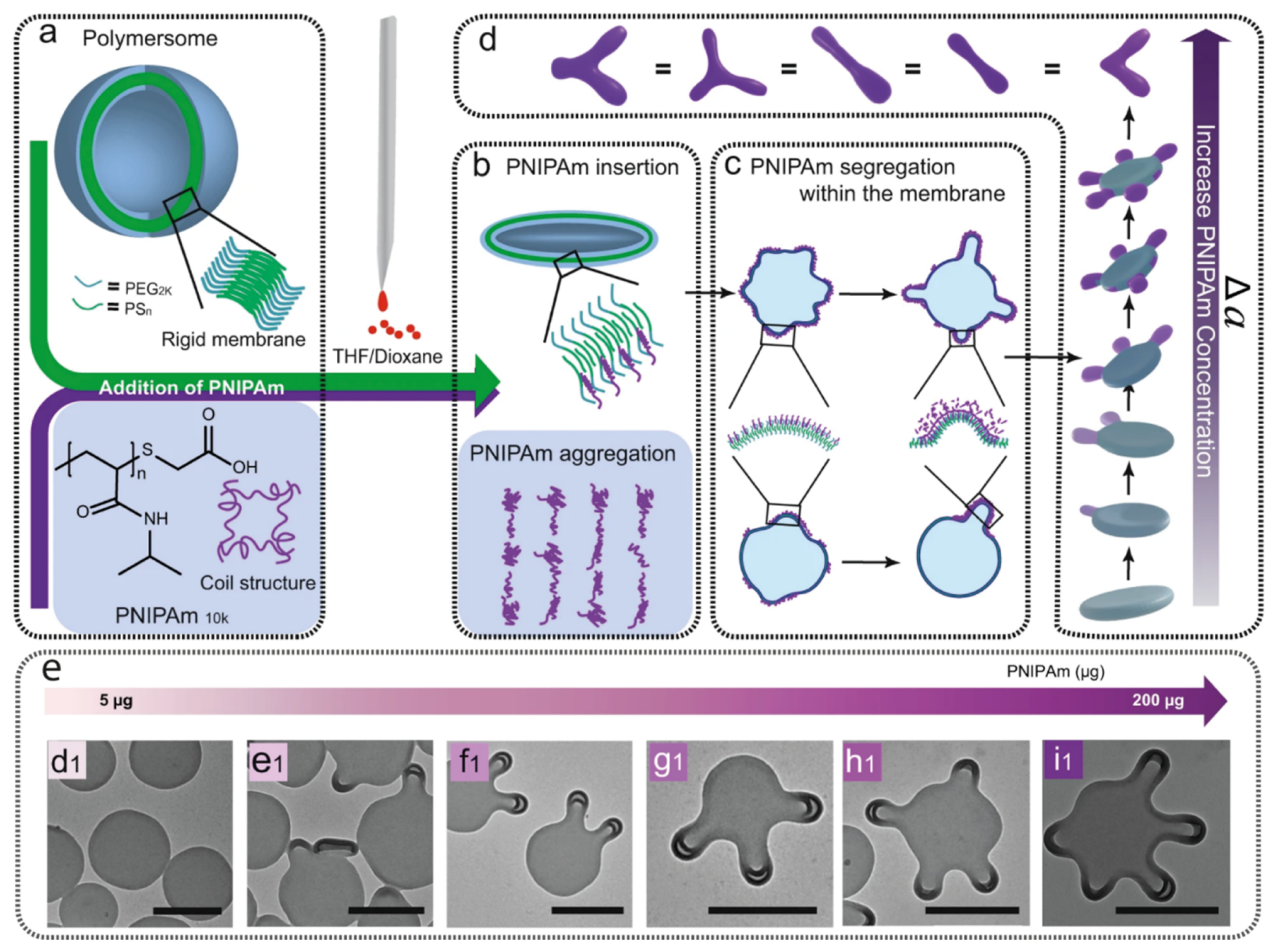
Sequential coupling of the oblate and prolate pathways by polymer insertion. (a, b) Shape transformation of the vesicle to the disc through the gradual addition of organic solvent (THF:dioxane = 4:1 v/v) into its aqueous dispersion (oblate pathway). (c, d) Local curvature generation on the disc by cononsolvent-induced insertion and segregation of poly(*N*-isopropylacrylamide) (prolate pathway). (e) Shapes obtained with increasing amounts of poly(*N*-isopropylacrylamide). Reproduced with permission from ref (57). Copyright 2021 Springer Nature.

Recently, we reported the dynamic insertion of poly(*N*-isopropylacrylamide) (PNIPAm) into the vesicular membrane caused by the cononsolvency phenomenon.^57^ The control over the interaction between PNIPAm and the vesicular membrane enables the sequential coupling of the oblate and prolate pathways (Figure 8). PNIPAm is initially dissolved in the aqueous dispersion of PEG-*b*-PS vesicles. Upon the gradual injection of organic solvent (THF:dioxane = 4:1 v/v), the vesicle is first transformed into a disc through the oblate pathway due to the osmotic gradient caused by different solvent compositions across the vesicular membrane (Figure 8a). By further increasing the ratio of organic solvent, PNIPAm would go through a hydrophilic to hydrophobic transition. Under these conditions, PNIPAm would be inserted into the outer layer of the vesicular membrane due to the hydrophobic effect (Figure 8b). The subsequent segregation of PNIPAm on the vesicular membrane would generate disparity in the surface area between the outer and the inner layers, which facilitates the formation of tubular arms along the rim of the disc through the prolate pathway (Figure 8c). Upon increasing the amount of PNIPAm, the number of tubular arms rises due to the enhanced coverage of the vesicular membrane until the disc disappears and multiarmed tubules are formed (Figure 8d and 8e). The dependence of shape transformation on PNIPAm demonstrates that the shift from the oblate to the prolate pathway is induced by polymer insertion. The sequential coupling of the oblate and prolate pathways successfully transforms vesicles into advanced architectures, such as a disc with tubular arms and multiarmed tubules.

Furthermore, salt is used to modulate the interaction between PNIPAm and the vesicular membrane.^58^ NaNO_3_ and PNIPAm were added into the aqueous dispersion of PEG-*b*-PS vesicles at the same time. NO_3_^–^ was proposed to bind to PNIPAm. Upon the injection of organic solvent, instead of forming aggregation, PNIPAm would stay as single-swelled chains. This would favor the insertion of PNIPAm into the vesicular membrane, increasing the number of tubular arms along the rim. As-obtained disc with tubular arms can transform along the oblate pathway to a stomatocyte with tubular arms through the continuous injection of organic solvent. Overall, polymer insertion enables the sequential coupling of oblate and prolate pathways.

### 4.2 Sequential Coupling of the Prolate and Oblate Pathways by Osmotic Stress

**Figure 9:**
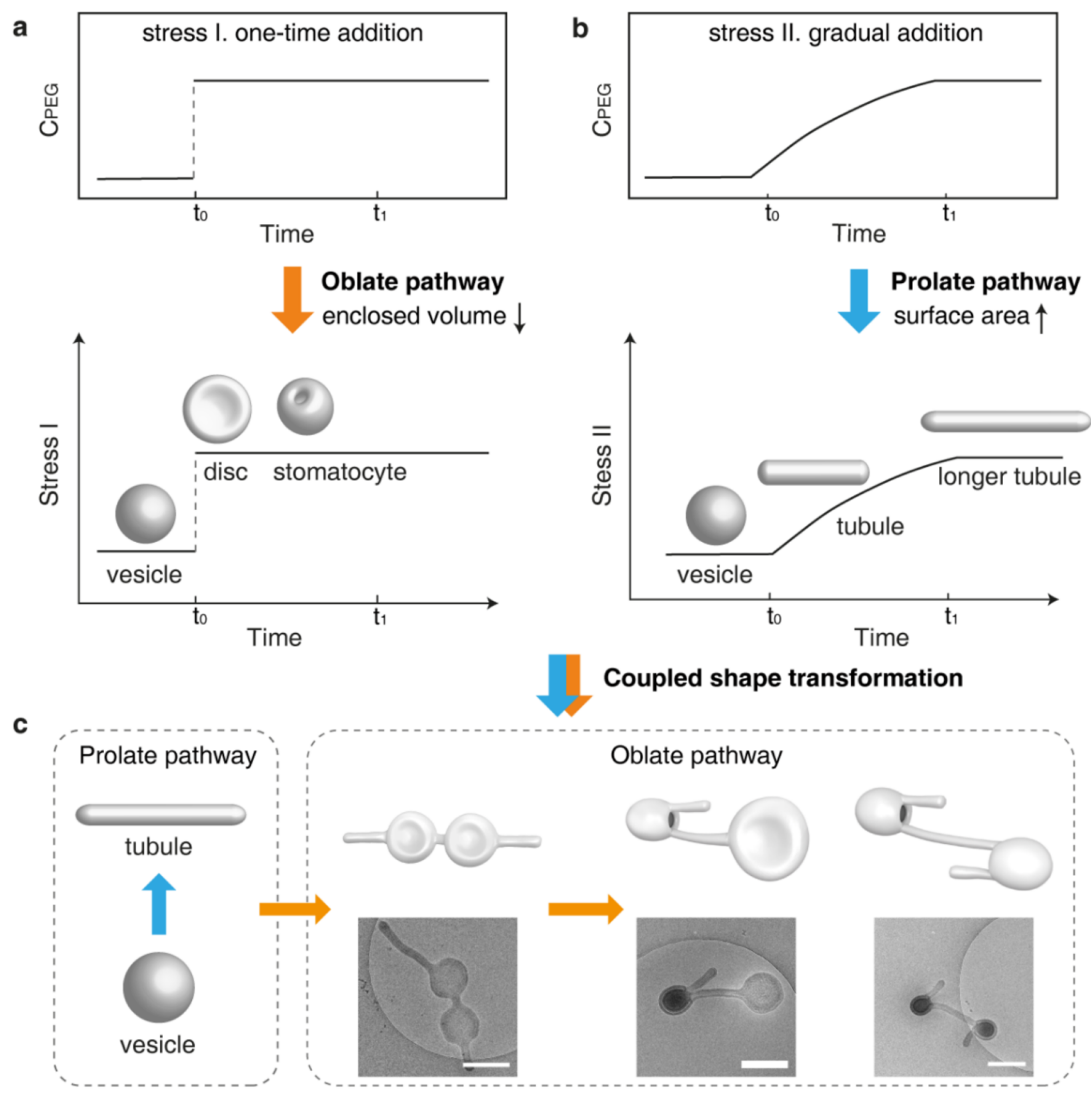
Sequential coupling of the prolate and oblate pathways by stress-dependent deformation of the vesicles. (a) Oblate pathway triggered by one-time addition of PEG (osmotic stress I). (b) Prolate pathway triggered by gradual addition of PEG (osmotic stress II). (c) Sequential coupling of prolate and oblate pathways by manipulating the osmotic stress. Reproduced with permission from ref (59). Copyright 2024 WILEY.

Alternative to the coupled shape transformation discussed in section 4.1, we also realize the sequential coupling of prolate and oblate pathways by manipulating the osmotic stress (Figure 9).^59^ In a previous study, it is believed that the vesicular membrane needs to be modified to trigger different deformation pathways.^56^ We recently found that osmotic stress can also control the deformation pathway of polymer vesicles. Specifically, osmotic shock caused by the one-time addition of PEG (osmotic stress I) would induce the shape transformation along the oblate pathway (Figure 9a). The mild osmotic stress caused by the gradual addition of PEG (osmotic stress II) would induce the shape transformation along the prolate pathway (Figure 9b). With the stress-dependent shape transformation in hand, we started to explore the sequential coupling of the prolate pathway and oblate pathway (Figure 9c). The vesicle is first transformed into a tubule along the prolate pathway by applying osmotic stress II. The obtained tubule is then transformed into a tubule with disc and stomatocyte through the localized deformation along the oblate pathway by applying osmotic stress I. The sequential coupling of prolate and oblate pathways enables us to access compartment networks of stomatocytes, which have not been accessed before. The coupling of basic deformation pathways presents new opportunities for the shape transformation of polymer vesicles. Currently, the coupled shape transformation is still at its initial stage. We envision that the investigation of the coupling of deformation pathways will advance the shape transformation of polymer vesicles from the trial-and-error stage to the computing stage.

## 5 Conclusion and Future Perspectives

In this Account, we introduced recent progress in the shape transformation of polymer vesicles. We first discussed the two basic deformation pathways of polymer vesicles: the oblate pathway and the prolate pathway. The methods used to trigger the two basic deformation pathways were included. Subsequently, we discussed the selectivity of the basic deformation pathways and current methods used to control the selectivity. Finally, we discussed the coupled deformation of polymer vesicles by focusing on the switch and coupling of two basic deformation pathways. We anticipate that our analysis of the deformation pathways will advance the understanding of the shape transformation of vesicles from the trial-and-error stage to the computing stage. The shape of nanoparticles has been shown to influence their circulation time, cellular uptake efficiency, and targeting capability when employed as drug carriers. The investigation of the shape transformation of polymer vesicles enables the generation of nanoparticles with diverse shapes while maintaining a consistent surface. This endeavor establishes a valuable database for elucidating the effects of nanoparticle shapes on their efficiency in drug delivery. Moreover, the expertise in the shape transformation of polymer vesicles may inspire the development of shape-morphing materials for cutting-edge technologies.

Since 1999, remarkable achievements have been made in the development of smart vesicular systems, aimed at emulating biological shape modulation. These advancements are driven by the synergy between active force generation and the passive materials’ properties, which jointly determine the shape transformation of polymer vesicles. To date, a variety of polymer vesicles, assembled from a diverse range of block polymers, have been utilized for the investigation of shape transformations triggered by various inputs (Table 1). According to the intermediate and final shapes, basic deformation pathways and coupled deformation pathways have been identified. The understanding of the deformation pathway represents a vital step toward the advent of the computing stage for the shape transformation of polymer vesicles, where desirable shapes for man-made applications, such as drug delivery, can be produced by adjusting the input parameter. However, several challenges still need to be overcome before the real advent of the computing stage.

### Development of the Shape Transformation Module

In the past two decades, significant efforts have been dedicated to studying the shape transformation of polymer vesicles. This has provided us with a large data set of deformation methods, deformation pathways, available shapes, and deformation mechanisms. To facilitate the advent of the computing stage, we believe the next step is to engineer shape transformation modules according to the data set. Each shape transformation module should be able to induce a basic deformation. Different shape transformation modules can be connected to produce the desirable shapes. This does not necessarily require new experiments but, more importantly, a modular perspective on existing data.

### Connection of Shape Transformation Modules

With the shape transformation modules in hand, we next need to investigate the connection of the shape transformation module. The connection efficiency and compatibility between different modules should be the focus. This can help us to redesign the shape transformation modules or build the connection rules for the shape transformation modules. The understanding of the deformation pathway can aid in the connection of shape transformation modules. The development of shape transformation modules and connection rules can help us to produce desirable shapes of polymer vesicles by manipulating the input parameter while not relying on trial and error. Moreover, artificial intelligence can be incorporated to facilitate the development of shape transformation modules and to predict the available pathway to a desired shape. This would greatly improve the efficiency of the design and production of new shapes and facilitate their application in the real world.

### Localized Deformation of Polymer Vesicles

Cells have the remarkable ability to generate positive curvature and negative curvature simultaneously across various regions of their membrane. Achieving such precise control remains a formidable challenge for polymer vesicles. Overcoming this challenge necessitates the creation of machinery capable of, at the very least, (1) inducing localized positive and negative curvature and (2) being interconnected to induce deformation concurrently. The advancement of synthetic machinery with the precision to induce localized deformations represents a significant step toward emulating cellular processes and enhancing our comprehension of cellular shape adaptation.

### Programmable Shape Transformation of Polymer Vesicles in Biological Environments

Polymer vesicles with adaptive shapes are highly pursued for smart nanomedicine. Currently, different stimuli including temperature, osmotic pressure, and magnetic field have been explored to induce the shape transformation of polymer vesicles. However, many of these deformations involve the use of organic solvents. Investigating the shape transformation of polymer vesicles in water represents a crucial direction. Several notable studies have already been published in this regard, but further efforts are necessary to enhance control over the shape transformation process.^43,60^ Additionally, the influence of biomolecules on the shapes of polymer vesicles remains unclear. Harnessing biological cues to modulate the shapes of polymer vesicles could be ground breaking. This approach could enable polymer vesicles to assume different shapes as they navigate different parts of the human body, facilitating their traversal through various biological barriers during drug delivery.
